# Neutralizing Antibody-Based Prevention of Cell-Associated HIV-1 Infection

**DOI:** 10.3390/v10060333

**Published:** 2018-06-18

**Authors:** Matthew S Parsons, Roger Le Grand, Stephen J Kent

**Affiliations:** 1 Department of Microbiology and Immunology, Peter Doherty Institute for Infection and Immunity, The University of Melbourne, Melbourne, Victoria 3000, Australia; skent@unimelb.edu.au; 2 CEA, Université Paris Sud 11, INSERM u1184, Immunology of Viral Infections and Autoimmune Diseases, IDMIT Department, IBFJ, Fontenay-aux-Roses 92265, France; roger.le-grand@cea.fr; 3 Melbourne Sexual Health Clinic and Infectious Disease Department, Alfred Hospital, Monash University Central Clinical School, Melbourne, Victoria 3053, Australia; 4 ARC Centre of Excellence in Convergent Bio-Nano Science and Technology, The University of Melbourne, Parkville, Victoria 3052, Australia

**Keywords:** HIV-1, cell-associated virus, broadly neutralizing antibody, simian–human immunodeficiency virus

## Abstract

Improved vaccine-mediated protection against HIV-1 requires a thorough understanding of the mode of HIV-1 transmission and how various immune responses control transmission. Cell-associated HIV-1 is infectious and contributes to HIV-1 transmission in humans. Non-human primate models of cell-associated SIV infection demonstrate that cell-associated SIV is more infectious than cell-free SIV. In a recently described chimeric simian–human immunodeficiency virus (SHIV) macaque model, it was demonstrated that an occult infection with cell-associated SHIV can be established that evades passive protection with a broadly neutralizing antibody (bnAb). Indeed, considerable in vitro data shows that bnAbs have less efficacy against cell-associated HIV-1 than cell-free HIV-1. Optimizing the protective capacity of immune responses such as bnAbs against cell-associated infections may be needed to maximize their protective efficacy.

## 1. Introduction

Immune-based prophylactics and/or vaccines are urgently needed to slow the spread of new HIV-1 infections. An ideal goal of such interventions is the establishment of antibodies that potently neutralize broad arrays of viral isolates, which are termed broadly neutralizing antibodies (bnAbs) [1]. Many bnAbs have now been isolated from HIV-1-infected donors [2]. BnAbs target several key vulnerable regions of the HIV-1 envelope, including the CD4 binding site [3], membrane proximal external region [4], trimer apex [5], gp120–gp41 interface [6], and high-mannose patch [7]. As well as neutralizing HIV-1 in vitro, bnAbs inhibit HIV-1 infection in mucosal explants [8]. Importantly, systemic or mucosal passive immunization of macaques with bnAbs protects against in vivo cell-free simian/human immunodeficiency virus (SHIV) challenges [9,10,11,12,13,14,15]. BnAbs also show potent efficacy as therapeutics, reducing SHIV viremia in macaques and HIV-1 viremia in humans [16,17]. However, it is important to note that immune escape can evolve to therapeutically administered bnAbs [17].

The success of bnAb passive immunization in animal models has motivated attempts to passively establish these antibodies in humans at risk of HIV-1 infection. Currently, two ongoing clinical trials (NCT02568215 and NCT02716675) are assessing the efficacy of a passively administered CD4 binding site bnAb, VRC01, to prevent HIV-1 acquisition in high-risk participants [18]. Additionally, there is much desire to design vaccine constructs that are capable of eliciting bnAb production in vaccine recipients. While attempts to induce bnAbs through immunization have not generated successful outcomes, there is hope that sequential immunization protocols might slowly shape bnAb precursors into potent neutralizing antibodies and reveal a path forward for inducing bnAbs by vaccination [19]. In the absence of vaccines that successfully elicit bnAbs, gene transfer using adeno-associated virus (AAV) vectors could represent a means of establishing these antibodies in individuals at risk of HIV-1 infection [20,21,22]. 

A potential impediment to the utility of bnAbs for preventing HIV-1 infection is the existence of HIV-1 as cell-associated virus (CAV) within infectious body fluids [23,24]. CAV is highly infectious in vitro [25] and in vivo [26]. Furthermore, semen-derived CAV is responsible for at least a proportion of new HIV-1 infections [27]. Early research into preventing infection following exposure to CAV assessed the protective capacity of antiviral T cells. An immunization study in macaques revealed that T cell immunity can confer protection from CAV exposure, but only in animals with a matched MHC-I allele [28]. Antibody-based immunity overcomes the issue of mismatched MHC-I between recipients of HIV-1 vaccines and the donors of HIV-1-infected CAV. However, implementing bnAbs to prevent HIV-1 infection following exposure to CAV is not without caveats. Importantly, much in vitro evidence suggests that CAV can evade neutralization by some bnAbs and/or is neutralized only with higher concentrations of bnAb [29,30,31,32,33,34,35], although the significance of these observations is understudied in animal models. We recently determined that the PGT121 bnAb provides macaques with partial protection from intravenous cell-associated SHIV challenge, and full protection from intravenous cell-free SHIV challenge [36]. Further studies will likely be highly informative for designing vaccines and/or immune-based prophylactics that are capable of robustly preventing infection with both cell-free virus and CAV.

This manuscript reviews the evidence of CAV involvement in viral transmission, as well as the capacity of CAV to evade antibody neutralization. We discuss non-human primate models of CAV transmission and their utility for assessing bnAb-based prevention of infection. Lastly, we examine opportunities for future research that will drive the optimization of bnAb prevention of CAV transmission, leading to the development of strategies to prevent infection by both cell-associated and cell-free virus. 

## 2. The “Trojan Horse” Hypothesis: Evidence for HIV-1 Transmission by CAV

Anderson and Yunis (1983) first proposed the hypothesis that a cell-associated pathogen contributes to the etiology of AIDS in 1983 [37]. This was a remarkably prescient publication, given that HIV-1 had not yet been defined as the causative agent of AIDS. The possibility of HIV-1 transmitting as CAV was later promoted by Levy (1988) in the context of CAV representing the principle means of transmission for several retroviruses, including human T cell leukemia virus, bovine leukemia virus, and Visna virus [38]. More recent discussions of the “Trojan horse” hypothesis have highlighted the accumulation of data from in vitro and ex vivo experiments, in vivo animal models, and human clinical studies that support a role for CAV in HIV-1 transmission [39,40,41,42,43]. Indeed, it is now clear that cell-associated HIV-1 transmits between cells, and is likely responsible for at least a proportion of new HIV-1 infections.

Much in vitro research has demonstrated that the transmission of HIV-1 between infected donor cells and uninfected target cells occurs across intercellular contact areas that are termed virological synapses (VS). The details of the VS, including the cell surface proteins involved and the involvement of the actin cytoskeleton in facilitating cell-to-cell transmission, have been reviewed elsewhere [44,45]. The characteristics of VS have been described between infected and uninfected T cells [46], as well as infected macrophages and uninfected T cells [30]. In general, the VS depends on the interaction of the HIV-1 envelope from the infected donor cell with CD4 on the uninfected target cell [46]. These interactions are supported by adhesion molecule interactions [47]. Once the VS is established, the virus is released toward the target cell and can establish infection through membrane fusion or endocytosis [45]. Compared with the direct infection of T cells by cell-free virus, HIV-1 transfer across VS is more efficient. Several potential reasons for the increased efficacy of cell-to-cell HIV-1 spread compared with cell-free infection have been discussed [25,39], including (i) the proximity of donor and target cells; (ii) the potential for the receptors involved in viral entry to cluster at the site of target/donor cell contact; and (iii) the increased multiplicity of infection present at a VS compared with cell-free spread. 

In addition to the transmission of HIV-1 from infected to uninfected cells, HIV-1 can be transmitted by non-infected cells harboring the virus to uninfected target cells. The donor cells for this type of infection include cell types that can gather the virus without necessarily becoming infected, such as dendritic cells. Such donor cells establish a synapse with target cells that is known as an infectious synapse (IS) [44,48,49]. The details of this mode of HIV-1 transmission have been reviewed elsewhere [50].

In a closer approximation of an HIV-1 transmission scenario, the infectiousness of cell-associated SIV and HIV-1 have also been evaluated in colon explant models derived from rhesus macaques and humans, respectively [26]. The results of these experiments not only demonstrated the infectious nature of CAV, but also suggest CAV to be more infectious than free virus. While the treatment of macaque colon explants with one milliliter of plasma from an SIV-infected macaque (corresponding to 2.3 × 10^3^ TCID_50_), serving as a source of cell-free virus, did not establish infection, treatment of explants with one million PBMC from an SIV-infected macaque (corresponding to 1.8 × 10^2^ TCID_50_), as a source of CAV, initiated infection. A similar experiment was also conducted using human colon explants exposed to cell-free HIV-1 or human CD4^+^ T-cells infected with HIV-1 in vitro. Five days following exposure, explants were digested and analyzed by flow cytometry for HIV-1-infected CD3^+^CD8^-^ T cells. Robust infections were initiated within the colon explants by CAV. Indeed, 27% of CD3^+^CD8^-^ T cells were infected following exposure to a CAV dose corresponding to 3.5 × 10^3^ TCID_50_. To obtain a similar level of T cell infection within explants following cell-free virus exposure, a 10 times higher dose of cell-free virus was required. These data not only highlight an increased infectiousness of CAV compared with cell-free virus, but also point to the potential for CAV to contribute to HIV-1 transmission following sexual exposure. Indeed, as the authors highlighted, infected cells applied to mucosal explants can actively migrate across the epithelium and initiate infection. 

As reviewed by Anderson (2014), several studies have also assessed the ability of CAV to establish infections in female genital tract explants [39]. One study demonstrated that both cell-free virus and CAV establish infection across ectocervical explant tissue [51]. Another study demonstrated that semen-derived cells penetrate ectocervical epithelium, but become trapped within mucus at the endocervical surface [52]. 

In addition to in vitro and ex vivo data supporting the feasibility of CAV contributing to HIV-1 transmission, several in vivo studies demonstrate the possibility of CAV transmission. Indeed, animal studies in murine and non-human primate models have demonstrated that the administration of CAV by the intravenous, intrarectal, oral, or intravaginal routes can establish systemic infection [26,36,53,54,55,56,57,58,59]. Moench (2014) recently reviewed the use of humanized mice for assessing infection following CAV exposure [42]. Early studies of CAV transmission in humanized mice utilized the hu-PBL-SCID model. The hu-PBL-SCID model has been used to demonstrate that HIV-1 CAV can initiate infection following vaginal administration to Depo-Provera-treated animals [58,59]. Similarly, the humanized BLT mouse model has been utilized to assess the infectiousness of CAV. Oral challenges of BLT mice with CAV initiated infections [57]. Non-human primate models of CAV infection are discussed in more detail later within this review (see section—Non-human primate models of CAV infection). 

**Figure 1:**
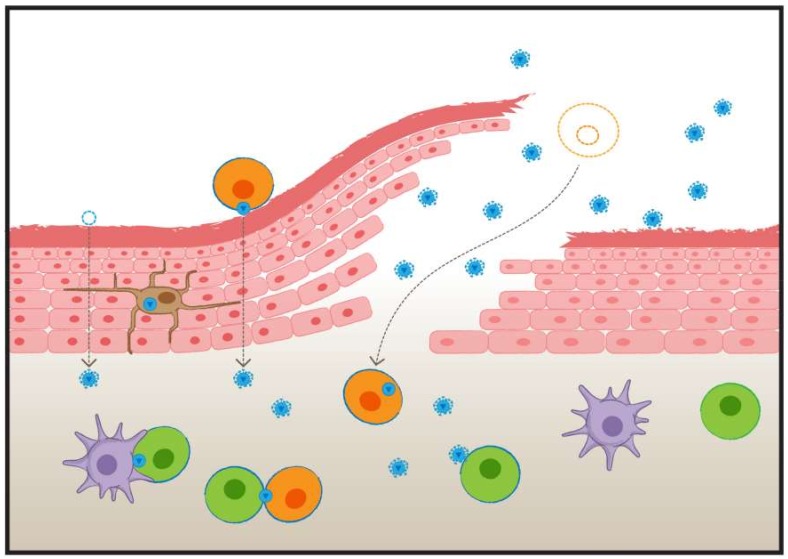
HIV-1 exposure at the vagina. HIV-1 enters the vagina as both cell-associated virus (CAV) and free virus. Both cell-associated and cell-free virus can migrate across an intact barrier. Antigen-presenting cells within the epithelium may assist migration by picking up free virus or by carrying virus transferred to them by infected “Trojan horse” cells. Both cell-free virus and CAV may also cross the vaginal epithelium by entering through abrasions. Once across the vaginal epithelium, newly infected cells can be established by the transfer of virus from infected allogeneic “Trojan horse” cells (Orange) to uninfected autologous T cells (Green), or the infection of autologous T cells by free virus particles. Autologous dendritic cells (Purple) may also gather virus and infect autologous cells across infectious synapses. Blue borders indicate cells that are infected or in the process of becoming infected. The dotted orange cell reflects an infected allogeneic “Trojan horse” cell prior to crossing the epithelium through an abrasion.

Lastly, several clinical studies point towards a role for CAV in HIV-1 transmission. Ronen et al. (2015) and Milligan and Overbaugh (2014) recently reviewed the evidence of CAV contributing to human HIV-1 transmission via sexual exposure or mother-to-child transmission [41,43]. With regards to sexual transmission, Zhu et al. (1996) noted that CAV and cell-free virus within semen were genetically distinct [27]. They assessed the virus in the blood of newly infected individuals from known transmission couples and observed that this virus often most closely resembled the CAV from the semen of the transmitting partner (i.e., three out of five cases). A similar study in men who have sex with men observed that the transmitted virus most closely resembled the cell-free virus from the semen of the transmitting partner in all cases (i.e., 6/6) [60]. Collectively, these results likely reflect a situation where both CAV and cell-free virus contribute to HIV-1 transmission following sexual exposure. Potential means of sexual transmission of CAV, using the female genital tract as a model, are presented in Figure 1. The transmission of HIV-1 through breastfeeding has been linked to both cell-free virus and CAV within breast milk. Indeed, the presence of CAV within breast milk correlates with transmission [24]. Furthermore, increasing levels of CAV and cell-free virus within breast milk have been tied to increasing risk of infection [61,62]. Importantly, increased levels of CAV are associated with increased infection risk, even after correcting for cell-free virus [63]. Lastly, there is evidence that CAV contributes to HIV-1 transmission through breastfeeding early postpartum (i.e., first six weeks) but is likely a less important contributor to transmission from six months onward [64]. Similar to HIV-1 transmission following sexual exposure, the collective evidence from studies of HIV-1 transmission through breastfeeding implies that both CAV and cell-free virus are responsible for establishing new infections.

## 3. Does Transmission of HIV-1 as CAV Facilitate Viral Evasion of BnAbs?

A potential ramification of HIV-1 being transmittable as both cell-free virus and CAV is the possibility that free virions and CAV are differentially susceptible to immune-based interventions that are designed to prevent infection. As mentioned earlier, an ideal goal of HIV-1 vaccine development is the induction of antibodies with properties similar to bnAbs. It has been hypothesized that the existence of HIV-1 as CAV might represent a mechanism through which virus can evade antibody-based immunity [40]. Studies assessing the capacity of bnAbs to inhibit cell-to-cell HIV-1 spread have produced conflicting results, with some studies demonstrating bnAbs to inhibit viral spread and others reporting a decreased efficacy of bnAbs to inhibit cell-to-cell spread compared with cell-free infection [25,29,30,31,32,33,34,35,65,66]. These divergent results have largely been attributed to the varied experimental systems applied by different research groups [49]. Despite these mixed results, the weight of evidence suggests that bnAbs exhibit a reduced efficacy against CAV. This is commonly observed as higher 50% inhibitory concentrations (IC_50_).

The reduced efficacy of bnAb inhibition of CAV compared with cell-free viral spread has been noted in studies assessing cell-to-cell spread across VS between infected and uninfected T cells [31], as well as those formed between infected macrophages and uninfected T cells [30]. There is great diversity in the ability of bnAbs to inhibit the cell-to-cell spread of HIV-1. This diversity is observed between bnAbs of different specificities [29], and has been noted to depend on the combination of bnAb and the viral strain being assessed [35]. Furthermore, certain combinations of bnAbs more efficiently inhibit the cell-to-cell spread of HIV-1 than single bnAbs [32]. 

In addition to demonstrations of increased IC_50_ values for the bnAb inhibition of CAV compared with the cell-free virus, Li et al. (2017) observed that the maximum neutralization of CAV by bnAbs is lower than the maximum neutralization of the cell-free virus [33]. Indeed, the maximum neutralization of cell-free spread by bnAbs was usually over 90%, while the maximum neutralization of CAV at saturating concentrations of bnAbs dropped to as low as 36%. This pattern of incomplete neutralization of cell-to-cell spread of HIV-1 was noted across a range of bnAbs. Furthermore, incomplete neutralization was primarily observed against transmitter/founder viruses compared with lab-adapted strains. The authors suggested that the different maximum neutralization of lab-adapted and transmitter/founder CAV could be due to the presence of different envelope conformations on cells infected with each type of virus. 

In addition to cell surface envelope conformations, several other aspects of cell-to-cell spread have been discussed as potential contributors to the blunting of bnAb efficacy against CAV [49]. These factors include the increased MOI of cell-to-cell spread and steric hindrances that prevent bnAbs from accessing their cognate epitopes. The relative role for each of these variables in reducing bnAb efficacy against CAV in in vitro assays remains poorly defined. Lastly, it is important to note that the relevance of in vitro studies demonstrating a reduced efficacy of bnAbs against CAV to the in vivo efficacy of the same bnAbs is an understudied area. 

## 4. Non-Human Primate Models of CAV Infection

Robust in vivo systems of viral infection are required to assess the effectiveness of putative prophylactic interventions against HIV-1, such as bnAbs. Indeed, non-human primate models of HIV-1 infection utilizing SHIV have been repeatedly employed to assess the capacity of bnAbs to prevent infection following cell-free viral challenge [9,10,11,12,13,14,15]. While most non-human primate models of HIV-1 infection have utilized free virus, there has been some effort to create robust models of infections initiated by exposure to CAV.

Early attempts to initiate infections by exposure to CAV focused on intravenous inoculation with infected cells. Such challenges established SIV infections in rhesus macaques [28,56]. The efficacy of viral transmission following the intravenous inoculation of CAV is robust, with one study reporting as few as two SIV-infected cells being sufficient to initiate an infection [56].

In addition to establishing the infectiousness of CAV following intravenous inoculation, effort has been directed toward establishing models of infections initiated by CAV across the mucosal surface at the female reproduction tract. Initial attempts to infect rhesus macaques with SIV by vaginal exposure to CAV were entirely unsuccessful [56]. Furthermore, mucosal challenge of chimpanzees with HIV-1 CAV was only partially successful at establishing infection [53]. More recently, two independent groups demonstrated infection of cynomolgus macaques following vaginal exposure to SIV CAV. Kaizu et al. (2006) reported that repeated exposure to CAV (i.e., 5000–500,000 in vitro infected PBMC) was sufficient to infect all macaques with experimentally induced genital ulceration and a proportion of control macaques lacking genital ulcers [54]. Salle et al. (2010) challenged macaques vaginally with splenocytes derived from two SIV-infected macaques [55]. Challenges with 10^7^ splenocytes from the first donor macaque infected three out of three animals. The same challenge dose with splenocytes from the second donor macaque infected one out of two animals. In addition to confirming that vaginal exposure to CAV can establish systemic infection, Salle et al. (2010) traced the migration of the inoculated CAV within challenged animals. Intriguingly, the inoculated cells traversed the vaginal epithelium, and could be identified in proximal and distal lymphoid tissues within one to two days. 

Complementing efforts to establish non-human primate models of CAV transmission at the female reproductive tract, SIV infection following rectal challenge with CAV was recently reported in rhesus macaques. Kolodkin-Gal et al. (2013) performed a repeated low-dose rectal challenge with equivalent 92 TCID_50_ doses of cell-free virus and CAV in rhesus macaques [26]. Following two challenges, three out of five CAV exposed macaques were infected. None of the four animals exposed to cell-free virus became infected over four challenges. These data indicate that CAV can establish infections rectally, and might be more infectious at this site of exposure than free virions. 

An important component of the discussed studies assessing the infectiousness of CAV following vaginal or rectal exposure are the viral inocula. As noted by the authors of these studies [26,54,55], the number of cells, particularly infected cells, that were required to initiate infection following mucosal challenge fell within the range of that expected within the average ejaculate of an HIV-1-infected male. This is a major strength of mucosal CAV models, and adds credence to CAV as a robust means of viral transmission.

## 5. Establishment of SHIV Models of CAV Infection and Assessment of BnAb-Conferred Protection

Non-human primate models of SIV CAV infection are an important proof of concept that exposure to CAV initiates infection. Furthermore, these models can serve as tools to evaluate the mechanisms through which CAV establishes infection. However, the assessment of anti-HIV-1 bnAb-conferred protection from infection following CAV exposure requires viruses expressing the HIV-1 envelope, such as SHIV. We recently developed a SHIV-based CAV challenge model in pigtailed macaques [36]. This system was utilized to screen the PGT121 bnAb for its capacity to block infection following CAV exposure.

To establish a macaque SHIV CAV challenge model, we focused on developing a system that consistently established robust infections, making the effects of passively transferred bnAbs on infectivity easy to interpret. To meet this goal, we opted to establish an intravenous challenge system with SHIV_SF162P3_ CAV. Briefly, a SHIV_SF162P3_ CAV challenge stock was generated by harvesting the spleen of an acutely SHIV_SF162P3_-infected macaque at a time of high plasma viremia and cryopreserving splenocytes. Next, in order to establish the infectiousness of the CAV challenge stock, approximately 25 × 10^6^ splenocytes were administered to five animals infused with a human IgG1 isotype control antibody or no antibody. Control animals exhibited high viral loads one week post-challenge, and developed plasma anti-gp41 antibodies three weeks post-challenge. The utilized challenge dose contained approximately 1000 animal infectious doses and 3 × 10^3^ viral DNA copies per 1 × 10^6^ cells. In contrast to the robust infection observed in control animals, an infusion of 1mg/kg of the PGT121 bnAb conferred partial protection from infection. Complete protection was noted in three PGT121-infused macaques. Two PGT121-infused macaques exhibited a short delay in peak viremia, and one passively immunized animal did not exhibit viremia until eight weeks post-challenge.

The patterns of protection from SHIV_SF162P3_ CAV observed in PGT121-treated macaques were partly explained by the plasma levels of antibodies recorded in these animals. Indeed, the two animals with the short delay prior to peak viremia had low suboptimal concentrations of plasma PGT121 at the time of challenge. However, the animal exhibiting delayed viremia at eight weeks post-challenge presented similar levels and dynamics of antibody waning as the three animals that were completely protected from CAV challenge. We hypothesized that this animal likely retained a latent virus that remained hidden until PGT121 levels waned, allowing for viral replication and the establishment of a systemic infection. 

Our model of SHIV_SF162P3_ CAV infection of pigtailed macaques revealed two key observations. First, we noted that the PGT121 bnAb can protect animals from infection following CAV exposure. While three out of six animals did become infected despite passive immunization with PGT121, an assessment of plasma levels of PGT121 suggested that this was partly a result of insufficient initial concentrations of antibody or antibody waning. Consistent with in vitro data on CAV neutralization by bnAbs, it may be that higher concentrations of bnAbs are required to protect against CAV [33]. Secondly, our study highlighted the possibility for CAV challenge to establish ‘occult’ infections that remain hidden until protective immunity wanes to suboptimal levels. Similar observations have been made in animals exposed to high-dose cell-free virus following treatment with antiretroviral therapy-based pre-exposure prophylactics (PrEP) [67,68]. Although we are uncertain if the ‘occult’ infection that we observed in an animal following CAV exposure was a ramification of the high-dose challenge employed, we have recently discussed this observation in detail, and noted the need for clinical trials of PrEP and bnAb-based passive immunization to carefully watch for such cases in HIV-1 exposed humans [69]. 

## 6. Opportunities to Optimize BnAb Prevention of CAV Transmission

The accumulation of evidence that CAV contributes to HIV-1 transmission highlights the need for strategies to optimize bnAb-mediated protection from infection following CAV exposure. The establishment of non-human primate CAV challenge models provides ideal systems for testing such strategies. In addition to experiments in non-human primate CAV challenge models, attempts to optimize bnAb-conferred protection from infection following CAV exposure will benefit from complimentary in vitro assays.

As discussed earlier, numerous independent research groups have attempted to design in vitro experimental systems to assess the capacity of bnAbs to inhibit CAV [25,29,30,31,32,33,34,35,65,66]. These experimental systems are highly variable in terms of the viruses utilized, as well as the target and donor cells employed. Further variability between these experimental systems has been introduced in how the authors have attempted to distinguish infections resulting from cell-free virus and CAV. Likely resulting from this high degree of experimental variation, attempts to assess the capacity of bnAbs to inhibit CAV have produced divergent results. At present, there is little evidence regarding which in vitro results most closely reflect the capacity of bnAbs to prevent infection following in vivo CAV exposure. As such, there is a need for a systematic study of in vitro assays of bnAb inhibition of cell-to-cell virus spread conducted in parallel with bnAb passive immunization studies in non-human primate CAV challenge models. These experiments will be important for validating which in vitro assay(s) can predict the capacity of bnAbs to prevent infection following CAV exposure in vivo.

In addition to establishing in vitro assays that accurately predict the capacity of BnAbs to inhibit cell-to-cell viral spread in vivo, there is a need to refine in vivo non-human primate CAV challenge models to more closely reflect common routes of HIV-1 transmission between humans. This would involve designing mucosal models of SHIV CAV challenge, which utilize infected cell types reflective of those found in mucosal secretions. Establishing such models might be facilitated by the observation that infected macrophages and CD4^+^ T cells are present in cynomolgus macaque semen following SIV infection [70]. Such cells could be applied to mucosal sites, including the rectum and vagina, to attempt to initiate new infections. This could be attempted with doses reflecting naturally occurring cell numbers, or the cells could be pooled from several ejaculates and used in a high-dose challenge system. Creating large pools of semen-derived leukocytes provides a means of overcoming potential differences in provirus content between ejaculates. 

Another means of improving non-human primate CAV challenge models to more closely reflect HIV-1 transmission between humans is to utilize SHIV that express envelopes from transmitter/founder HIV-1 strains. The most commonly utilized SHIV express envelopes from viruses derived from chronically infected donors. Envelopes from viruses isolated during chronic infection may not accurately portray the role that transmitter/founder envelopes play in establishing infection. In terms of CAV, transmitter/founder viruses are potentially highly significant, as these HIV-1 strains are incompletely neutralized by bnAbs [33]. Recently, SHIV expressing transmitter/founder virus envelopes have been discussed [71,72]. These viruses are ideal for implementation in non-human primate CAV challenge models. 

Lastly, non-human primate CAV challenge models are ideal for identifying the features of bnAbs that are required for optimal protection from infection. Indeed, these models can serve to identify if certain bnAb specificities are more ideal than others for preventing infection following CAV exposure, if combinations of bnAbs can provide more robust protection than single antibodies, and/or if bnAbs require the capacity to mediate non-neutralizing Fc-dependent functions to protect against infection. Given that Fc-dependent functions have been shown to be important for bnAb-conferred protection from cell-free virus in non-human primates and preventing virus entry in murine models [10,73], it seems likely that these functions might contribute to protection from CAV. Following exposure to CAV, there is a need to eliminate the infected cells within the inoculum, as well as any autologous cells that become infected. An infection of autologous cells following cell-free virus exposure has been noted to occur despite passive immunization with the PGT121 bnAb [74]. This phenomenon is likely to occur at a significantly higher magnitude in animals exposed to CAV, as CAV might be incompletely neutralized [33]. As such, we predict that bnAb-conferred protection from infection following CAV exposure involves Fc-dependent antibody functions. Improving the Fc-dependent functions of bnAbs may improve their capacity to protect against CAV. Improving the capacity of the CD4 binding site bnAb b12 to trigger ADCC did not improve protection from cell-free virus [75]. However, we suggest that bnAbs with improved Fc functions should be revisited for studies assessing protection from CAV, given its cell-based nature.

## 7. Conclusions

Many retroviruses can be transmitted from cell to cell, and HIV-1, SIV, and chimeric SHIV are also readily transmitted as CAV. Immune-based protection from CAV, particularly by neutralizing antibodies, may be more difficult to achieve than immune-based protection from cell-free virus. This has major implications for the rational design of vaccine strategies to combat HIV-1. We speculate that the dose and Fc-mediated functions of bnAbs can be optimized to improve the protective efficacy of bnAbs against cell-associated HIV-1.

## References

[1] Haynes B.F., Moody M.A., Alam M., Bonsignori M., Verkoczy L., Ferrari G., Gao F., Tomaras G.D., Liao H.X., Kelsoe G. (2014). Progress in HIV-1 vaccine development. J. Allergy Clin. Immunol..

[2] McCoy L.E., Burton D.R. (2017). Identification and specificity of broadly neutralizing antibodies against HIV. Immunol. Rev..

[3] Zhou T., Georgiev I., Wu X., Yang Z.Y., Dai K., Finzi A., Kwon Y.D., Scheid J.F., Shi W., Xu L. (2010). Structural basis for broad and potent neutralization of HIV-1 by antibody VRC01. Science.

[4] Huang J., Ofek G., Laub L., Louder M.K., Doria-Rose N.A., Longo N.S., Imamichi H., Bailer R.T., Chakrabarti B., Sharma S.K. (2012). Broad and potent neutralization of HIV-1 by a gp41-specific human antibody. Nature.

[5] Walker L.M., Phogat S.K., Chan-Hui P.Y., Wagner D., Phung P., Goss J.L., Wrin T., Simek M.D., Fling S., Mitcham J.L. (2009). Broad and potent neutralizing antibodies from an African donor reveal a new HIV-1 vaccine target. Science.

[6] Blattner C., Lee J.H., Sliepen K., Derking R., Falkowska E., de la Pena A.T., Cupo A., Julien J.P., van Gils M., Lee P.S. (2014). Structural delineation of a quaternary, cleavage-dependent epitope at the gp41-gp120 interface on intact HIV-1 Env trimers. Immunity.

[7] Walker L.M., Huber M., Doores K.J., Falkowska E., Pejchal R., Julien J.P., Wang S.K., Ramos A., Chan-Hui P.Y., Moyle M. (2011). Broad neutralization coverage of HIV by multiple highly potent antibodies. Nature.

[8] Cheeseman H.M., Olejniczak N.J., Rogers P.M., Evans A.B., King D.F., Ziprin P., Liao H.X., Haynes B.F., Shattock R.J. (2017). Broadly Neutralizing Antibodies Display Potential for Prevention of HIV-1 Infection of Mucosal Tissue Superior to That of Nonneutralizing Antibodies. J. Virol.

[9] Gautam R., Nishimura Y., Pegu A., Nason M.C., Klein F., Gazumyan A., Golijanin J., Buckler-White A., Sadjadpour R., Wang K. (2016). A single injection of anti-HIV-1 antibodies protects against repeated SHIV challenges. Nature.

[10] Hessell A.J., Hangartner L., Hunter M., Havenith C.E., Beurskens F.J., Bakker J.M., Lanigan C.M., Landucci G., Forthal D.N., Parren P.W. (2007). Fc receptor but not complement binding is important in antibody protection against HIV. Nature.

[11] Hessell A.J., Rakasz E.G., Poignard P., Hangartner L., Landucci G., Forthal D.N., Koff W.C., Watkins D.I., Burton D.R. (2009). Broadly neutralizing human anti-HIV antibody 2G12 is effective in protection against mucosal SHIV challenge even at low serum neutralizing titers. PLoS Pathog..

[12] Moldt B., Rakasz E.G., Schultz N., Chan-Hui P.Y., Swiderek K., Weisgrau K.L., Piaskowski S.M., Bergman Z., Watkins D.I., Poignard P. (2012). Highly potent HIV-specific antibody neutralization in vitro translates into effective protection against mucosal SHIV challenge in vivo. Proc. Natl. Acad. Sci. USA.

[13] Parren P.W., Marx P.A., Hessell A.J., Luckay A., Harouse J., Cheng-Mayer C., Moore J.P., Burton D.R. (2001). Antibody protects macaques against vaginal challenge with a pathogenic R5 simian/human immunodeficiency virus at serum levels giving complete neutralization in vitro. J. Virol..

[14] Veazey R.S., Shattock R.J., Pope M., Kirijan J.C., Jones J., Hu Q., Ketas T., Marx P.A., Klasse P.J., Burton D.R. (2003). Prevention of virus transmission to macaque monkeys by a vaginally applied monoclonal antibody to HIV-1 gp120. Nat. Med..

[15] Moog C., Dereuddre-Bosquet N., Teillaud J.L., Biedma M.E., Holl V., Van Ham G., Heyndrickx L., Van Dorsselaer A., Katinger D., Vcelar B. (2014). Protective effect of vaginal application of neutralizing and nonneutralizing inhibitory antibodies against vaginal SHIV challenge in macaques. Mucosal Immunol..

[16] Barouch D.H., Whitney J.B., Moldt B., Klein F., Oliveira T.Y., Liu J., Stephenson K.E., Chang H.W., Shekhar K., Gupta S. (2013). Therapeutic efficacy of potent neutralizing HIV-1-specific monoclonal antibodies in SHIV-infected rhesus monkeys. Nature.

[17] Caskey M., Schoofs T., Gruell H., Settler A., Karagounis T., Kreider E.F., Murrell B., Pfeifer N., Nogueira L., Oliveira T.Y. (2017). Antibody 10-1074 suppresses viremia in HIV-1-infected individuals. Nat. Med..

[18] Gilbert P.B., Juraska M., deCamp A.C., Karuna S., Edupuganti S., Mgodi N., Donnell D.J., Bentley C., Sista N., Andrew P. (2017). Basis and Statistical Design of the Passive HIV-1 Antibody Mediated Prevention (AMP) Test-of-Concept Efficacy Trials. Stat. Commun Infect. Dis.

[19] Escolano A., Steichen J.M., Dosenovic P., Kulp D.W., Golijanin J., Sok D., Freund N.T., Gitlin A.D., Oliveira T., Araki T. (2016). Sequential Immunization Elicits Broadly Neutralizing Anti-HIV-1 Antibodies in Ig Knockin Mice. Cell.

[20] Balazs A.B., Chen J., Hong C.M., Rao D.S., Yang L., Baltimore D. (2011). Antibody-based protection against HIV infection by vectored immunoprophylaxis. Nature.

[21] Gardner M.R., Kattenhorn L.M., Kondur H.R., von Schaewen M., Dorfman T., Chiang J.J., Haworth K.G., Decker J.M., Alpert M.D., Bailey C.C. (2015). AAV-expressed eCD4-Ig provides durable protection from multiple SHIV challenges. Nature.

[22] Saunders K.O., Wang L., Joyce M.G., Yang Z.Y., Balazs A.B., Cheng C., Ko S.Y., Kong W.P., Rudicell R.S., Georgiev I.S. (2015). Broadly Neutralizing Human Immunodeficiency Virus Type 1 Antibody Gene Transfer Protects Nonhuman Primates from Mucosal Simian-Human Immunodeficiency Virus Infection. J. Virol..

[23] Ho D.D., Schooley R.T., Rota T.R., Kaplan J.C., Flynn T., Salahuddin S.Z., Gonda M.A., Hirsch M.S. (1984). HTLV-III in the semen and blood of a healthy homosexual man. Science.

[24] Van de Perre P., Simonon A., Hitimana D.G., Dabis F., Msellati P., Mukamabano B., Butera J.B., Van Goethem C., Karita E., Lepage P. (1993). Infective and anti-infective properties of breastmilk from HIV-1-infected women. Lancet.

[25] Martin N., Welsch S., Jolly C., Briggs J.A., Vaux D., Sattentau Q.J. (2010). Virological synapse-mediated spread of human immunodeficiency virus type 1 between T cells is sensitive to entry inhibition. J. Virol..

[26] Kolodkin-Gal D., Hulot S.L., Korioth-Schmitz B., Gombos R.B., Zheng Y., Owuor J., Lifton M.A., Ayeni C., Najarian R.M., Yeh W.W. (2013). Efficiency of cell-free and cell-associated virus in mucosal transmission of human immunodeficiency virus type 1 and simian immunodeficiency virus. J. Virol..

[27] Zhu T., Wang N., Carr A., Nam D.S., Moor-Jankowski R., Cooper D.A., Ho D.D. (1996). Genetic characterization of human immunodeficiency virus type 1 in blood and genital secretions: Evidence for viral compartmentalization and selection during sexual transmission. J. Virol..

[28] Heeney J.L., van Els C., de Vries P., ten Haaft P., Otting N., Koornstra W., Boes J., Dubbes R., Niphuis H., Dings M. (1994). Major histocompatibility complex class I-associated vaccine protection from simian immunodeficiency virus-infected peripheral blood cells. J. Exp. Med..

[29] Abela I.A., Berlinger L., Schanz M., Reynell L., Gunthard H.F., Rusert P., Trkola A. (2012). Cell-cell transmission enables HIV-1 to evade inhibition by potent CD4bs directed antibodies. PLoS Pathog..

[30] Duncan C.J., Williams J.P., Schiffner T., Gartner K., Ochsenbauer C., Kappes J., Russell R.A., Frater J., Sattentau Q.J. (2014). High-multiplicity HIV-1 infection and neutralizing antibody evasion mediated by the macrophage-T cell virological synapse. J. Virol..

[31] Durham N.D., Yewdall A.W., Chen P., Lee R., Zony C., Robinson J.E., Chen B.K. (2012). Neutralization resistance of virological synapse-mediated HIV-1 Infection is regulated by the gp41 cytoplasmic tail. J. Virol..

[32] Gombos R.B., Kolodkin-Gal D., Eslamizar L., Owuor J.O., Mazzola E., Gonzalez A.M., Korioth-Schmitz B., Gelman R.S., Montefiori D.C., Haynes B.F. (2015). Inhibitory Effect of Individual or Combinations of Broadly Neutralizing Antibodies and Antiviral Reagents against Cell-Free and Cell-to-Cell HIV-1 Transmission. J. Virol..

[33] Li H., Zony C., Chen P., Chen B.K. (2017). Reduced Potency and Incomplete Neutralization of Broadly Neutralizing Antibodies against Cell-to-Cell Transmission of HIV-1 with Transmitted Founder Envs. J. Virol..

[34] Malbec M., Porrot F., Rua R., Horwitz J., Klein F., Halper-Stromberg A., Scheid J.F., Eden C., Mouquet H., Nussenzweig M.C. (2013). Broadly neutralizing antibodies that inhibit HIV-1 cell to cell transmission. J. Exp. Med..

[35] Reh L., Magnus C., Schanz M., Weber J., Uhr T., Rusert P., Trkola A. (2015). Capacity of Broadly Neutralizing Antibodies to Inhibit HIV-1 Cell-Cell Transmission Is Strain- and Epitope-Dependent. PLoS Pathog..

[36] Parsons M.S., Lloyd S.B., Lee W.S., Kristensen A.B., Amarasena T., Center R.J., Keele B.F., Lifson J.D., LaBranche C.C., Montefiori D. (2017). Partial efficacy of a broadly neutralizing antibody against cell-associated SHIV infection. Sci. Transl. Med..

[37] Anderson D.J., Yunis E.J. (1983). “Trojan Horse” leukocytes in AIDS. N. Engl. J. Med..

[38] Levy J.A. (1988). The transmission of AIDS: The case of the infected cell. JAMA.

[39] Anderson D.J. (2014). Modeling mucosal cell-associated HIV type 1 transmission in vitro. J. Infect. Dis.

[40] Anderson D.J., Politch J.A., Nadolski A.M., Blaskewicz C.D., Pudney J., Mayer K.H. (2010). Targeting Trojan Horse leukocytes for HIV prevention. AIDS.

[41] Milligan C., Overbaugh J. (2014). The role of cell-associated virus in mother-to-child HIV transmission. J. Infect. Dis..

[42] Moench T.R. (2014). Cell-associated transmission of HIV type 1 and other lentiviruses in small-animal models. J. Infect. Dis..

[43] Ronen K., Sharma A., Overbaugh J. (2015). HIV transmission biology: Translation for HIV prevention. AIDS.

[44] Ospina Stella A., Turville S. (2018). All-Round Manipulation of the Actin Cytoskeleton by HIV. Viruses.

[45] Sattentau Q.J. (2010). Cell-to-Cell Spread of Retroviruses. Viruses.

[46] Jolly C., Kashefi K., Hollinshead M., Sattentau Q.J. (2004). HIV-1 cell to cell transfer across an Env-induced, actin-dependent synapse. J. Exp. Med..

[47] Jolly C., Mitar I., Sattentau Q.J. (2007). Adhesion molecule interactions facilitate human immunodeficiency virus type 1-induced virological synapse formation between T cells. J. Virol..

[48] Rodriguez-Plata M.T., Puigdomenech I., Izquierdo-Useros N., Puertas M.C., Carrillo J., Erkizia I., Clotet B., Blanco J., Martinez-Picado J. (2013). The infectious synapse formed between mature dendritic cells and CD4(+) T cells is independent of the presence of the HIV-1 envelope glycoprotein. Retrovirology.

[49] Schiffner T., Sattentau Q.J., Duncan C.J. (2013). Cell-to-cell spread of HIV-1 and evasion of neutralizing antibodies. Vaccine.

[50] McDonald D. (2010). Dendritic Cells and HIV-1 Trans-Infection. Viruses.

[51] Collins K.B., Patterson B.K., Naus G.J., Landers D.V., Gupta P. (2000). Development of an in vitro organ culture model to study transmission of HIV-1 in the female genital tract. Nat. Med..

[52] Maher D., Wu X., Schacker T., Horbul J., Southern P. (2005). HIV binding, penetration, and primary infection in human cervicovaginal tissue. Proc. Natl. Acad. Sci. USA.

[53] Girard M., Mahoney J., Wei Q., van der Ryst E., Muchmore E., Barre-Sinoussi F., Fultz P.N. (1998). Genital infection of female chimpanzees with human immunodeficiency virus type 1. AIDS Res. Hum. Retrovir..

[54] Kaizu M., Weiler A.M., Weisgrau K.L., Vielhuber K.A., May G., Piaskowski S.M., Furlott J., Maness N.J., Friedrich T.C., Loffredo J.T. (2006). Repeated intravaginal inoculation with cell-associated simian immunodeficiency virus results in persistent infection of nonhuman primates. J. Infect. Dis..

[55] Salle B., Brochard P., Bourry O., Mannioui A., Andrieu T., Prevot S., Dejucq-Rainsford N., Dereuddre-Bosquet N., Le Grand R. (2010). Infection of macaques after vaginal exposure to cell-associated simian immunodeficiency virus. J. Infect. Dis..

[56] Sodora D.L., Gettie A., Miller C.J., Marx P.A. (1998). Vaginal transmission of SIV: Assessing infectivity and hormonal influences in macaques inoculated with cell-free and cell-associated viral stocks. AIDS Res. Hum. Retrovir..

[57] Wahl A., Swanson M.D., Nochi T., Olesen R., Denton P.W., Chateau M., Garcia J.V. (2012). Human breast milk and antiretrovirals dramatically reduce oral HIV-1 transmission in BLT humanized mice. PLoS Pathog..

[58] Di Fabio S., Giannini G., Lapenta C., Spada M., Binelli A., Germinario E., Sestili P., Belardelli F., Proietti E., Vella S. (2001). Vaginal transmission of HIV-1 in hu-SCID mice: A new model for the evaluation of vaginal microbicides. AIDS.

[59] Khanna K.V., Whaley K.J., Zeitlin L., Moench T.R., Mehrazar K., Cone R.A., Liao Z., Hildreth J.E., Hoen T.E., Shultz L. (2002). Vaginal transmission of cell-associated HIV-1 in the mouse is blocked by a topical, membrane-modifying agent. J. Clin. Investig..

[60] Butler D.M., Delport W., Kosakovsky Pond S.L., Lakdawala M.K., Cheng P.M., Little S.J., Richman D.D., Smith D.M. (2010). The origins of sexually transmitted HIV among men who have sex with men. Sci. Transl. Med..

[61] Koulinska I.N., Villamor E., Chaplin B., Msamanga G., Fawzi W., Renjifo B., Essex M. (2006). Transmission of cell-free and cell-associated HIV-1 through breast-feeding. J. Acquir. Immune Defic. Syndr..

[62] Rousseau C.M., Nduati R.W., Richardson B.A., Steele M.S., John-Stewart G.C., Mbori-Ngacha D.A., Kreiss J.K., Overbaugh J. (2003). Longitudinal analysis of human immunodeficiency virus type 1 RNA in breast milk and of its relationship to infant infection and maternal disease. J. Infect. Dis..

[63] Rousseau C.M., Nduati R.W., Richardson B.A., John-Stewart G.C., Mbori-Ngacha D.A., Kreiss J.K., Overbaugh J. (2004). Association of levels of HIV-1-infected breast milk cells and risk of mother-to-child transmission. J. Infect. Dis..

[64] Ndirangu J., Viljoen J., Bland R.M., Danaviah S., Thorne C., Van de Perre P., Newell M.L. (2012). Cell-free (RNA) and cell-associated (DNA) HIV-1 and postnatal transmission through breastfeeding. PLoS ONE.

[65] Massanella M., Puigdomenech I., Cabrera C., Fernandez-Figueras M.T., Aucher A., Gaibelet G., Hudrisier D., Garcia E., Bofill M., Clotet B. (2009). Antigp41 antibodies fail to block early events of virological synapses but inhibit HIV spread between T cells. AIDS.

[66] McCoy L.E., Groppelli E., Blanchetot C., de Haard H., Verrips T., Rutten L., Weiss R.A., Jolly C. (2014). Neutralisation of HIV-1 cell-cell spread by human and llama antibodies. Retrovirology.

[67] Andrews C.D., Yueh Y.L., Spreen W.R., St Bernard L., Boente-Carrera M., Rodriguez K., Gettie A., Russell-Lodrigue K., Blanchard J., Ford S. (2015). A long-acting integrase inhibitor protects female macaques from repeated high-dose intravaginal SHIV challenge. Sci. Transl. Med..

[68] Kovarova M., Council O.D., Date A.A., Long J.M., Nochi T., Belshan M., Shibata A., Vincent H., Baker C.E., Thayer W.O. (2015). Nanoformulations of Rilpivirine for Topical Pericoital and Systemic Coitus-Independent Administration Efficiently Prevent HIV Transmission. PLoS Pathog..

[69] Parsons M.S., Cromer D., Davenport M.P., Kent S.J. (2018). HIV Reactivation after Partial Protection by Neutralizing Antibodies. Trends Immunol..

[70] Bernard-Stoecklin S., Gommet C., Corneau A.B., Guenounou S., Torres C., Dejucq-Rainsford N., Cosma A., Dereuddre-Bosquet N., Le Grand R. (2013). Semen CD4+ T cells and macrophages are productively infected at all stages of SIV infection in macaques. PLoS Pathog..

[71] Asmal M., Luedemann C., Lavine C.L., Mach L.V., Balachandran H., Brinkley C., Denny T.N., Lewis M.G., Anderson H., Pal R. (2015). Infection of monkeys by simian-human immunodeficiency viruses with transmitted/founder clade C HIV-1 envelopes. Virology.

[72] Del Prete G.Q., Ailers B., Moldt B., Keele B.F., Estes J.D., Rodriguez A., Sampias M., Oswald K., Fast R., Trubey C.M. (2014). Selection of unadapted, pathogenic SHIVs encoding newly transmitted HIV-1 envelope proteins. Cell. Host Microbe.

[73] Bournazos S., Klein F., Pietzsch J., Seaman M.S., Nussenzweig M.C., Ravetch J.V. (2014). Broadly neutralizing anti-HIV-1 antibodies require Fc effector functions for in vivo activity. Cell.

[74] Liu J., Ghneim K., Sok D., Bosche W.J., Li Y., Chipriano E., Berkemeier B., Oswald K., Borducchi E., Cabral C. (2016). Antibody-mediated protection against SHIV challenge includes systemic clearance of distal virus. Science.

[75] Moldt B., Shibata-Koyama M., Rakasz E.G., Schultz N., Kanda Y., Dunlop D.C., Finstad S.L., Jin C., Landucci G., Alpert M.D. (2012). A nonfucosylated variant of the anti-HIV-1 monoclonal antibody b12 has enhanced FcgammaRIIIa-mediated antiviral activity in vitro but does not improve protection against mucosal SHIV challenge in macaques. J. Virol..

